# AM-37 and ST-36 Are Small Molecule Bombesin Receptor Antagonists

**DOI:** 10.3389/fendo.2017.00176

**Published:** 2017-07-21

**Authors:** Terry W. Moody, Nicole Tashakkori, Samuel A. Mantey, Paola Moreno, Irene Ramos-Alvarez, Marcello Leopoldo, Robert T. Jensen

**Affiliations:** ^1^Department of Health and Human Services, National Cancer Institute, Center for Cancer Research, Bethesda, MD, United States; ^2^National Institute of Diabetes, Digestive and Kidney Disease, Digestive Diseases Branch, Bethesda, MD, United States; ^3^Dipartimento di Farmacia, Scienze del Farmaco, Università degli Studi di Bari Aldo Moro, Bari, Italy

**Keywords:** small molecule antagonists, GRPR, NMBR, bombesin receptor subtype-3, lung cancer

## Abstract

While peptide antagonists for the gastrin-releasing peptide receptor (BB_2_R), neuromedin B receptor (BB_1_R), and bombesin (BB) receptor subtype-3 (BRS-3) exist, there is a need to develop non-peptide small molecule inhibitors for all three BBR. The BB agonist (BA)1 binds with high affinity to the BB_1_R, BB_2_R, and BRS-3. In this communication, small molecule BBR antagonists were evaluated using human lung cancer cells. AM-37 and ST-36 inhibited binding to human BB_1_R, BB_2_R, and BRS-3 with similar affinity (*K*i = 1.4–10.8 µM). AM-13 and AM-14 were approximately an order of magnitude less potent than AM-37 and ST-36. The ability of BA1 to elevate cytosolic Ca^2+^ in human lung cancer cells transfected with BB_1_R, BB_2_R, and BRS-3 was antagonized by AM-37 and ST-36. BA1 increased tyrosine phosphorylation of the EGFR and ERK in lung cancer cells, which was blocked by AM-37 and ST-36. AM-37 and ST-36 reduced the growth of lung cancer cells that have BBR. The results indicate that AM-37 and ST-36 function as small molecule BB receptor antagonists.

## Introduction

The bombesin (BB) family of peptides is biologically active in the central nervous system (CNS) and periphery. BB, a 14 amino acid peptide isolated from frog skin, has 9 of the 10 same C-terminal amino acids as does human gastrin-releasing peptide (GRP), a 27 amino acid peptide (1). GRP binds with high affinity to the BB_2_R, which regulates pruritus, lung development, and gastrin secretion. Neuromedin B (NMB) is a 10 amino acid peptide with 70% sequence homology to the C-terminal of BB. NMB binds with high affinity to the BB_1_R and causes satiety, hypothermia, and thyrotropin (TSH) secretion from the pituitary (2). BB receptor subtype-3 (BRS-3) is an orphan receptor with homology to the BB_1_R and BB_2_R, and binds the universal agonist, BB agonist (BA)1, with high affinity as does the BB_1_R and BB_2_R (3). Because BRS-3 knockout mice have impaired energy balance, glucose homeostasis, and increased body weight, BRS-3 agonists may function as satiety agents (4). In the CNS, GRP and NMB may act in a paracrine manner being released from brain neurons in the hypothalamus and dentate gyrus, respectively, activating BB_2_R and BB_1_R in adjacent cells (5).

In numerous cancers, including lung cancer, GRP and NMB function in an autocrine manner to stimulate cellular proliferation. Small cell lung cancer (SCLC), a neuroendocrine tumor, has high levels of GRP (6, 7). GRP is secreted from SCLC and binds to cell surface BB_2_R resulting in increased cellular proliferation (8). NMB is present in both SCLC and non-small cell lung cancer (NSCLC) cells, and after secretion it binds to cell surface BB_1_R stimulating proliferation (9). Because many lung cancer cells have BB_1_R, BB_2_R, and/or BRS-3 there is a need to develop antagonists that block all three receptors of the BB family.

The human BB_1_R, BB_2_R, and BRS-3 contain 390, 384, and 399 amino acids and have approximately 50% sequence homology. The BB_1_R, BB_2_R, and BRS-3 are members of the rhodopsin β group G protein-coupled receptors (GPCR) family, and they interact with Gq causing phosphatidylinositol (PI) turnover (10). PI-4,5-bisphosphate (PIP_2_) is metabolized to diacylglycerol, which activates protein kinase C and inositol-trisphosphate (IP_3_) which causes elevated cytosolic Ca^2+^. Neuropeptide receptors regulate the transactivation of the epidermal growth factor (EGF) receptor leading to NSCLC proliferation (11). The proliferation of NSCLC cells caused by BA1 can be inhibited by the tyrosine kinase inhibitor (TKI) gefitinib or BBR antagonists. The actions of BA1 on BB_1_R, BB_2_R, and BRS-3 are antagonized selectively by PD168368, PD176252, and Bantag-1, respectively (12).

In the present study, small molecules were synthesized and their ability to antagonize BB_1_R, BB_2_R, and BRS-3 in lung cancer cells evaluated. The results indicate that AM-37 and ST-36 are useful agents to inhibit the growth of NSCLC cells which have BB_1_R, BB_2_R, or BRS-3.

## Materials and Methods

### Cell Culture

Non-small cell lung cancer cell line NCI-H1299 (ATCC, Manassas, VA, USA) was stably transfected with BB_1_R, BB_2_R, and BRS-3. The transfected cells were grown in RPMI-1640 containing 10% fetal bovine serum (FBS) with 0.3 mg/ml geneticin (Invitrogen, Grand Island, NY, USA). The transfected cells, which contained approximately 100,000 receptors/cell, were weekly split using trypsin/EDTA (13). In addition, lung cancer cell lines NCI-H727, H1299, and H1975 were purchased from ATCC and cultured in RPMI-1640, which contained 10% FBS. The cell types were derived from different human biopsy specimens. These studies were approved by the NIDDK biospecimens and biosafety committees.

### Ligand Synthesis

**Figure 1:**
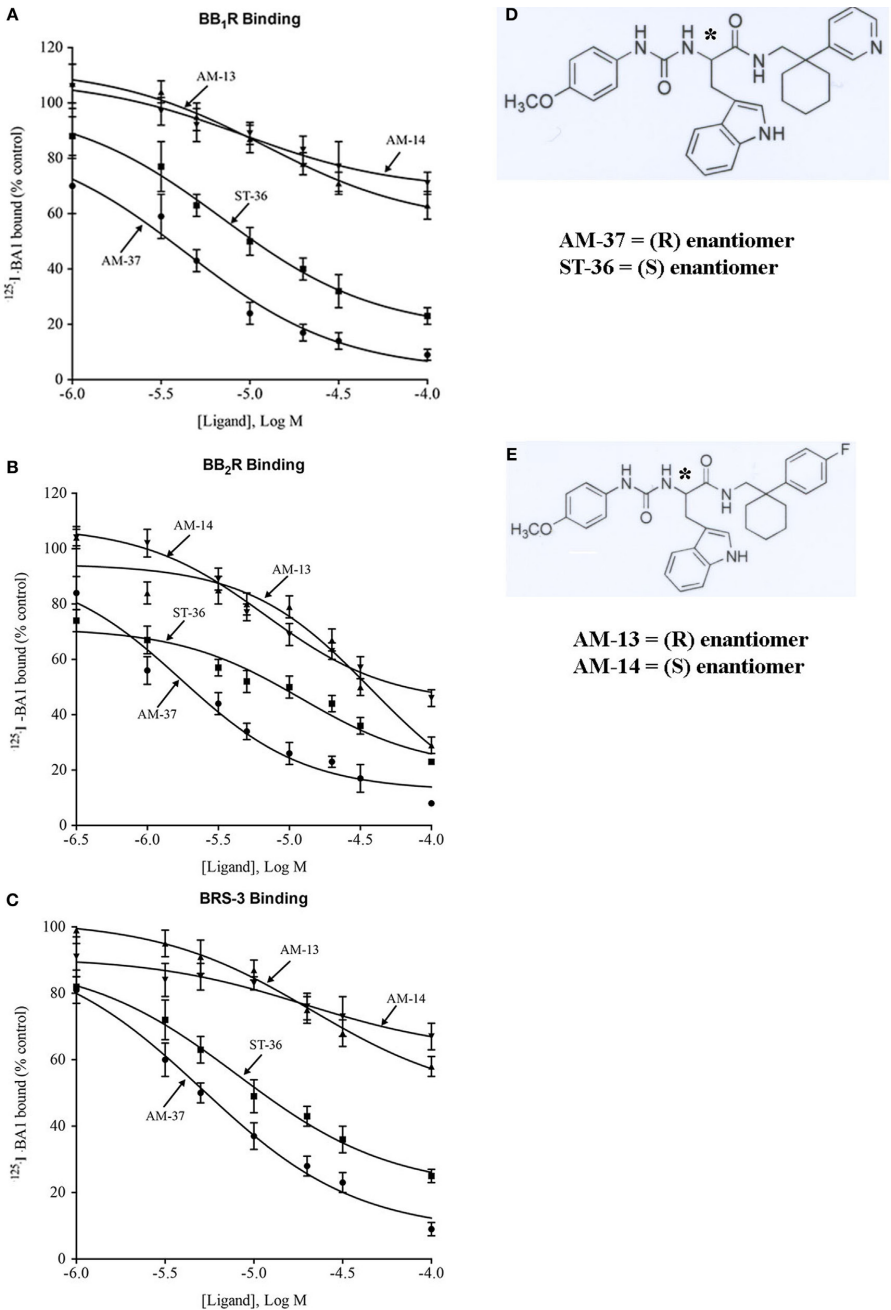
Binding. The ability of varying concentrations of AM-37 (●), ST-36 (■), AM-13 (▲), and AM-14 (▼) to inhibit specific ^125^I-BA1 binding was investigated using **(A)** BB_1_R-, **(B)** BB_2_R-, and **(C)** BRS-3-transfected NCI-H1299 cells. The mean value ± SD of three determinations each repeated in duplicate is shown. **(D)** The structure of AM-37 and ST-36 is shown. **(E)** The structure of AM-13 and AM-14 is shown; *indicates the optically active site.

The small molecules were synthesized as described previously (14). Figure 1D shows the structural formula of AM-37, (R)-3-(1H-indol-3-yl)-2-[3-(4-methoxyphenyl)ureido]-N-[[1-(3-pyridinyl)cyclohexyl]methyl]propanamide, and of its S-enantiomer ST-36. Figure 1E shows the structural formula of AM-13, (R)-N-[[1-(4-fluorophenyl)cyclohexyl]methyl]-3-(1H-indol-3-yl)-2-[3-(4-methoxyphenyl)ureido]propanamide, and its S-enantiomer AM-14. The molecular weight of AM-37 and ST-36 is 525.6 Da, whereas the molecular weight of AM-13 and AM-14 is 542.2 Da.

### Receptor Binding

The ability of AM-37, ST-36, AM-13, and AM-14 to inhibit specific ^125^I-BA1 binding to NSCLC cells transfected stably with BB_1_R, BB_2_R, and BRS-3 was investigated. NSCLC cells were placed in 24 well plates. When confluent, the cells were washed three times with PBS. The cells were incubated with binding buffer (PBS containing 0.25% bovine serum albumin and 0.025% bacitracin, Sigma-Aldrich, St. Louis, MO, USA). Various concentrations of AM-37, ST-36, AM-13, or AM-14 were added to the cells for 10 min, followed by 100,000 cpm of ^125^I-BA1 (0.16 nM) and incubated at 37°C for 30 min when equilibrium of binding was reached. The cells were rinsed three times with binding buffer for 2 min at 4°C. The cells that contained bound peptide dissolved in 0.2 N NaOH and counted in a Wallac 1470 γ-counter. The *K*_i_ was calculated as described (15).

### Cytosolic Ca^2+^

The ability of AM-37, ST-36, AM-13, and AM-14 to function as BBR antagonists was investigated. NSCLC cells transfected with BB_1_R, BB_2_R, and BRS-3 were harvested and loaded with Fura-2AM (Calbiochem, La Jolla, CA, USA) as described previously (16). The excitation ratio was determined at 340 and 380 nm with an emission wavelength of 510 nm. The lung cancer cellular calcium response was determined after the addition of AM-37, ST-36, AM-13, or AM-14 followed by 10 nM BA1.

### Tyrosine Phosphorylation

The tyrosine phosphorylation of the EGFR and ERK was investigated by western blot. NSCLC cells transfected with BB_1_R, BB_2_R, and BRS-3 were placed in 10 cm dishes. When the cells were confluent, they were placed in SIT medium (RPMI-1640 containing 3 × 10^−8^ M sodium selenite, 5 µg/ml bovine insulin, and 10 µg/ml apo-transferrin; Sigma-Aldrich, St. Louis, MO, USA) for 3 h. AM-37, ST-36, AM-13, or AM-14 were added for 30 min followed by 100 nM BA1 for 2 min. Cell extracts were made as described previously (16), and 600 µg of protein extract was immunoprecipitated with 4 µg anti-phosphotyrosine antibody (Becton Dickenson, USA). The immunoprecipitates were fractionated using a 4–20% polyacrylamide gel (Novex, San Diego, CA, USA). Proteins were transferred to a nitrocellulose membrane and incubated with 2 µg anti-EGFR or anti-ERK antibody (Cell Signaling Technologies, Danvers, MA, USA). After washing the blot, it was incubated with enhanced chemiluminescence detection reagent (Thermo Scientific) for 5 min and exposed to Biomax XAR film (Carestream, Rochester, NY, USA). The band intensity was determined using a Kodak image station 440 densitometer. Alternatively, 20 µg of protein extract was loaded onto polyacrylamide gels and after transfer to nitrocellulose, the blot was probed with anti-PY^1,068^-EGFR, anti-EGFR, anti-PY^204^ERK, or anti-ERK (Cell Signaling Technologies, Danvers, MA, USA).

### Proliferation

The proliferation of NSCLC cells was investigated using the 3-(4,5-demethylthiazol-2-yl)-2,3-diphenyl-2H-tetrazolium bromide (MTT) assay as described previously (16). NCI-H727, H1299, and H1975 cells were placed in SIT medium and varying concentration of AM-37, ST-36, AM-13, or AM-14 added. After 2 days, 0.1% MTT solution (15 µl) was added. After 4 h, DMSO (150 µl) was added and the absorbance at 570 nm was determined.

### Statistical Analysis

The results are expressed as the mean ± SD. Statistical significance of differences was performed by a one-way or two-way repeated measures of variance. The binding curves were drawn using PRISM.

## Results

### Receptor Binding

The ability of the small molecules to bind to BB_1_R, BB_2_R, and BRS-3 was investigated. AM-37 (R-enantiomer) inhibited specific ^125^I-BA1 binding to BB_1_R, BB_2_R, and BRS-3 in a dose-dependent manner with *K*_i_ values 3.6, 1.4, and 5.5 µM, respectively (Figure 1). ST-36 (S-enantiomer) inhibited specific ^125^I-BA1 binding to BB_1_R, BB_2_R, and BRS-3 with *K*i values of 7.9, 6.9, and 10.8 µM, respectively (Figure 1). In contrast, AM-13 (R-enantiomer) and AM-14 (S-enantiomer) inhibited specific ^125^I-BA1 binding to BB_1_R, BB_2_R, and BRS-3 with *K*_i_ > 20 µM. The results indicate that AM-37and ST-36 bind to BB_1_R, BB_2_R, and BRS-3 with greater affinity than does AM-13 and AM-14.

The specificity of binding was investigated. Table 1 shows that BA1 bound with high affinity (*K*_i_ = 0.002, 0.0005, and 0.004 µM) to BB_1_R, BB_2_R, and BRS-3. AM-37, ST-36, AM-13, and AM-14 inhibited specific ^125^I-BA1 binding (*K*_i_ = 1.4, 6.9, 27, and 45 µM) to BB_2_R. ST-36 inhibited specific ^125^I-BA1 binding (*K*_i_ = 7.9 and 10.8 µM) to BB_1_R and BRS-3, respectively. AM-13 and AM-14 bind with low affinity to BB_1_R and BRS-3 (*K*_i_ > 100 µM and >100 μM, respectively).

**Table 1:** Binding to lung cancer cells transfected with human bombesin receptors.

Ligand	*K*_i_, μM		
	BB_1_R	BB_2_R	BRS-3
BA1	0.002 ± 0.0002	0.0005 ± 0.0001	0.004 ± 0.0003
AM-37	3.6 ± 0.5	1.4 ± 0.2	5.5 ± 0.6
ST-36	7.9 ± 0.9	6.9 ± 0.3	10.8 ± 0.9
AM-13	>100	27 ± 4	>100
AM-14	>100	45 ± 8	>100

*The mean value ± SD of four determinations is indicated. The structure of bombesin agonist 1 is (D-Tyr^6^, β-Ala^11^, Phe^13^, Nle^14^)BB^6–14^*.

*BRS-3, bombesin receptor subtype-3*.

### Cytosolic Ca^2+^

**Figure 2:**
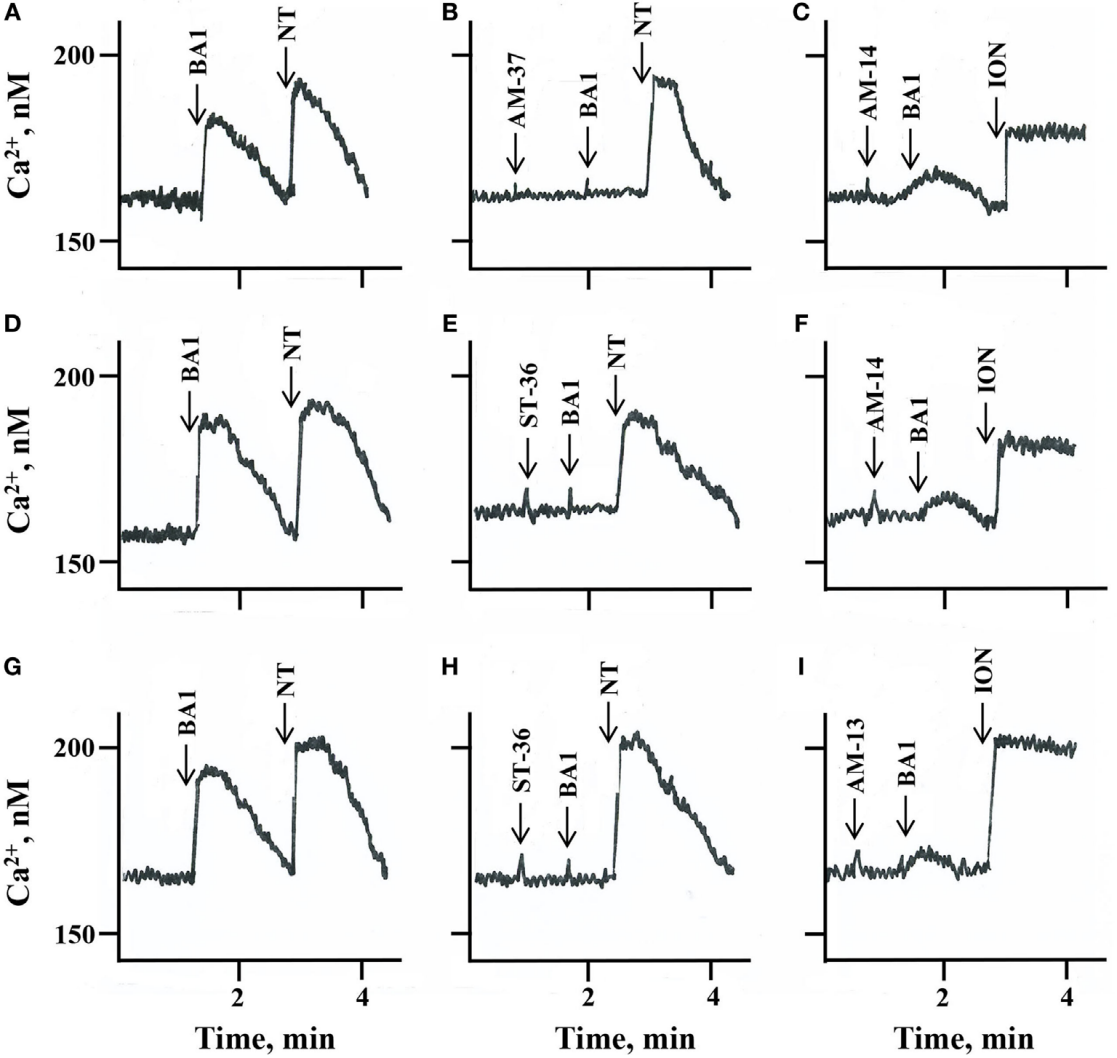
Cytosolic Ca^2+^. The ability of **(A)** 10 nM bombesin agonist 1 (BA)1 and 10 nM neurotensin (NT), **(B)** 30 µM AM-37 followed by 10 nM BA1 and 10 nM NT, and **(C)** 30 µM AM-14 followed by 10 nM BA1 and 5 µg/ml ionomycin (ION) to increase cytosolic Ca^2+^ was determined as a function of time after the addition to NCI-H1299 cells transfected with BB_1_R. The ability of **(D)** 10 nM BA1 and 10 nM NT, **(E)** 30 µM ST-36 followed by 10 nM BA1 and 10 nM NT, and **(F)** 30 µM AM-14 followed by 10 nM BA1 and 5 µg/ml ION to increase cytosolic Ca^2+^ was determined as a function of time after the addition to NCI-H1299 cells transfected with BB_2_R. The ability of **(G)** 10 nM BA1 and 10 nM NT, **(H)** 30 µM ST-36 followed by 10 nM BA1 and 10 nM NT, and **(I)** 30 µM AM-13 followed by 10 nM BA1 and 5 µg/ml ION to increase cytosolic Ca^2+^ was determined as a function of time after the addition to NCI-H1299 cells transfected with bombesin receptor subtype-3. This experiment is representative of three others.

The ability of the small molecules to function as BB_1_R, BB_2_R, and BRS-3 antagonists was investigated. Addition of 10 nM BA1 to NCI-H1299 cells transfected with BB_1_R increased the cytosolic Ca^2+^ from 160 to 178 nM within seconds (Figure 2A). The response was transient and returned to baseline after 1 min. Addition of 30 µM AM-37 to NCI-H1299 cells transfected with BB_1_R had no effect on the basal cytosolic Ca^2+^ but blocked the increase in cytosolic Ca^2+^ caused by BA1 (Figure 2B). Addition of 30 µM AM-14 had no effect of basal cytosolic Ca^2+^ but partially blocked the increase caused by 10 nM BA1 (Figure 2C). Table 2 shows that AM-37 and AM-14 significantly inhibited the ability of BA1 to increase cytosolic Ca^2+^ after addition to NCI-H1299 cells transfected with BB_1_R. Addition of 10 nM BA1 to NCI-H1299 cells transfected with BB_2_R increased the cytosolic Ca^2+^ from 160 to 186 nM (Figure 2D). Addition of 30 µM ST-36 to NCI-H1299 cells transfected with BB_2_R had no effect on the basal cytosolic Ca^2+^ but blocked the increase in cytosolic Ca^2+^ caused by BA1 (Figure 2E). Addition of 30 µM AM-14 had no effect of basal cytosolic Ca^2+^ but partially blocked the increase caused by 10 nM BA1 (Figure 2F). Table 2 shows that ST-36 and AM-14 significantly decreased the ability of 10 nM BA1 to elevate cytosolic Ca^2+^ in NCI-H1299 cells transfected with BB_2_R. Addition of 10 nM BA1 to NCI-H1299 cells transfected with BRS-3 increased the cytosolic Ca^2+^ from 170 to 194 nM (Figure 2G). Addition of 30 µM ST-36 to NCI-H1299 cells transfected with BRS-3 had no effect on the basal cytosolic Ca^2+^ but blocked the increase in cytosolic Ca^2+^ caused by BA1 (Figure 2H). Addition of 30 µM AM-13 had no effect of basal cytosolic Ca^2+^ but partially blocked the increase caused by 10 nM BA1 (Figure 2I). Table 2 shows that ST-36 and AM-13 significantly decreased the ability of 10 nM BA1 to elevate cytosolic Ca^2+^ in NCI-H1299 cells transfected with BRS-3. The results indicate that AM-37 and ST-36 are antagonists for BB_1_R, BB_2_R, and BRS-3. In contrast, AM-13 and AM-14 are weak antagonists for the BBR family.

**Table 2:** Increases in cytosolic Ca^2+^ using human lung cancer cells transfected with bombesin receptors.

Addition	Increase in cytosolic Ca^2+^, nM		
	BB_1_R	BB_2_R	BRS-3
BA1, 10 nM	18.5 ± 1.1	26.3 ± 1.7	24.4 ± 2.3
BA1 + AM-37, 30 µM	1 ± 0.6^a^	0^a^	0^a^
BA1 + ST-36, 30 µM	0^a^	0^a^	0^a^
BA1 + AM-13, 30 µM	7 ± 0.8^a^	5 ± 0.6^a^	2 ± 0.3^a^
BA1 + AM-14, 30 µM	6 + 0.6^a^	6 + 0.5^a^	3 + 0.4^a^

*The initial increase in the cytosolic Ca^2+^ after addition of BA1 to lung cancer cells containing BB_1_R, BB_2_R, or BRS-3 is indicated. Addition of small molecules significantly inhibited (*p* < 0.01;^a^ by ANOVA) the ability of BA1 to increase cytosolic Ca^2+^. This experiment is representative of three others*.

*BRS-3, bombesin receptor subtype-3, BA1, bombesin agonist 1*.

The specificity of AM-37, ST-36, AM-13, and AM-14 was investigated. 10 nM neurotensin (NT) or 5 µg/ml ionomycin (ION) strongly increased the cytosolic Ca^2+^ in NSCLC cells. AM-37 or ST-36 had no effect on the ability of NT to increase cytosolic Ca^2+^ in NSCLC cells. AM-13 or AM-14 had no effect on the ability of ION to increase Ca^2+^ in NSCLC cells. Therefore, AM-36 and ST-37 are antagonists for the BBR but not the NTR.

### Tyrosine Phosphorylation

**Figure 3:**
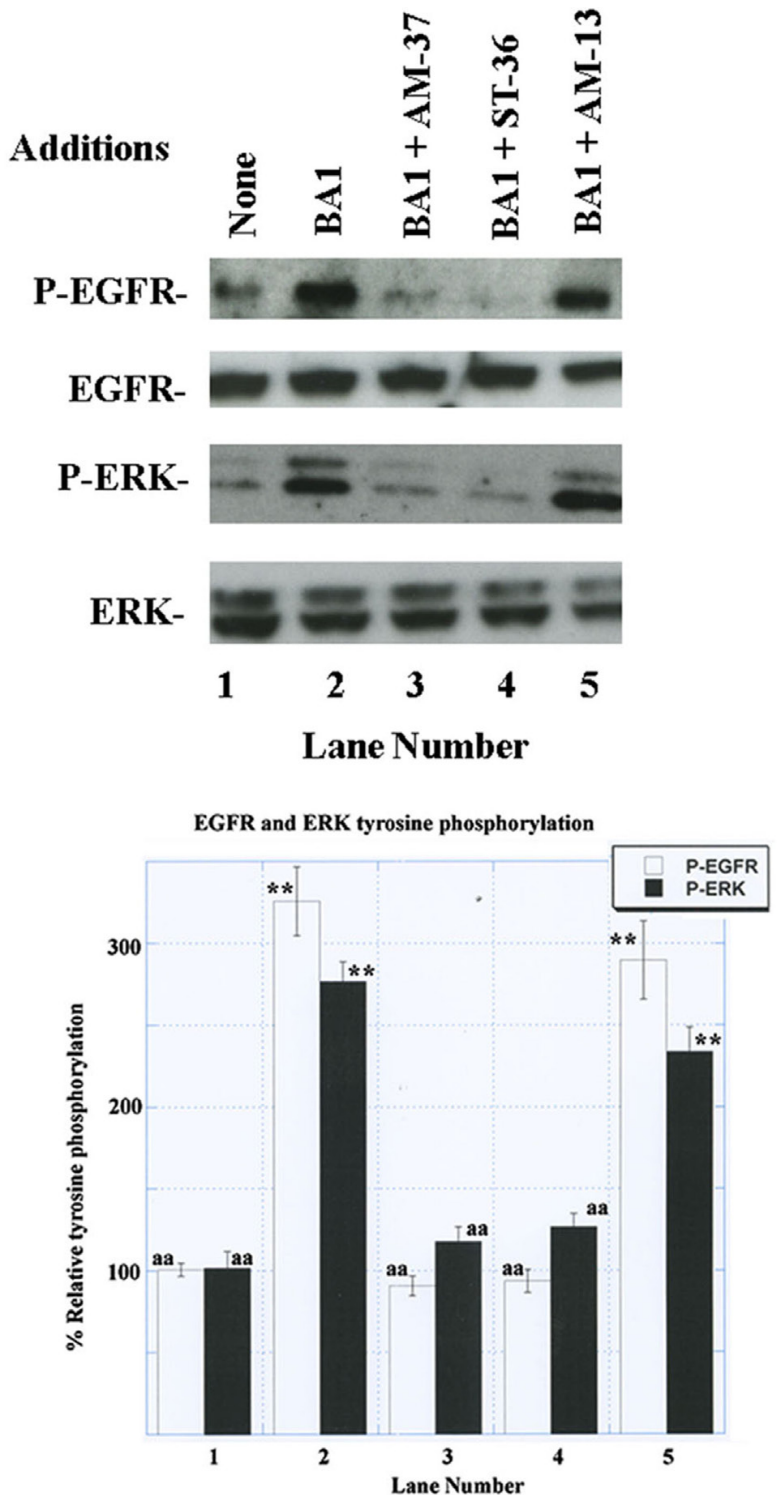
Western blot. (Top) The ability of 100 nM bombesin agonist (BA)1 to increase EGFR and ERK tyrosine phosphorylation was investigated using NCI-H1299 cells transfected with BB_2_R in the presence of 10 µM AM-37, 10 µM ST-36, and 10 µM AM-13. (Bottom) BA1 or BA1 plus AM-13 increased significantly EGFR and ERK tyrosine phosphorylation relative to the control, whereas total ERK and EGFR were unaltered; *p* < 0.01; ** by ANOVA. The control, BA1 + AM-37, and BA1 + ST-36 were significantly reduced relative to BA1; *p* < 0.01, ^aa^ by ANOVA. The experiment is representative of three others.

The ability of the small molecules to impair EGFR transactivation was investigated. Previously, we found that the BB_1_R and BRS-3 regulate EGFR tyrosine phosphorylation (13, 16). Figure 3 shows that addition of 100 nM BA1 to NCI-H1299 cells transfected with BB_2_R increased significantly the EGFR tyrosine phosphorylation to 326%. If the cells were pretreated with 10 µM AM-37 or ST-36, addition of BA1 had little effect. In contrast, if the cells were treated with 10 µM AM-13, BA1 increased strongly EGFR tyrosine phosphorylation. Similarly, BA1 addition to NCI-H1299 cells transfected with BB_2_R increased ERK tyrosine phosphorylation to 277%. This increase in ERK tyrosine phosphorylation was decreased significantly in the cells pretreated with AM-37 or ST-36 but not AM-13. Similarly, AM-14 had little effect on EGFR or ERK tyrosine phosphorylation (data not shown). The results indicate that AM-37 and ST-36 antagonize the ability of the BB_2_R to regulate tyrosine phosphorylation of the EGFR and ERK. Similar transactivation results were obtained for NSCLC cells transfected with BB_1_R or BRS-3 (data not shown).

### Proliferation

**Figure 4:**
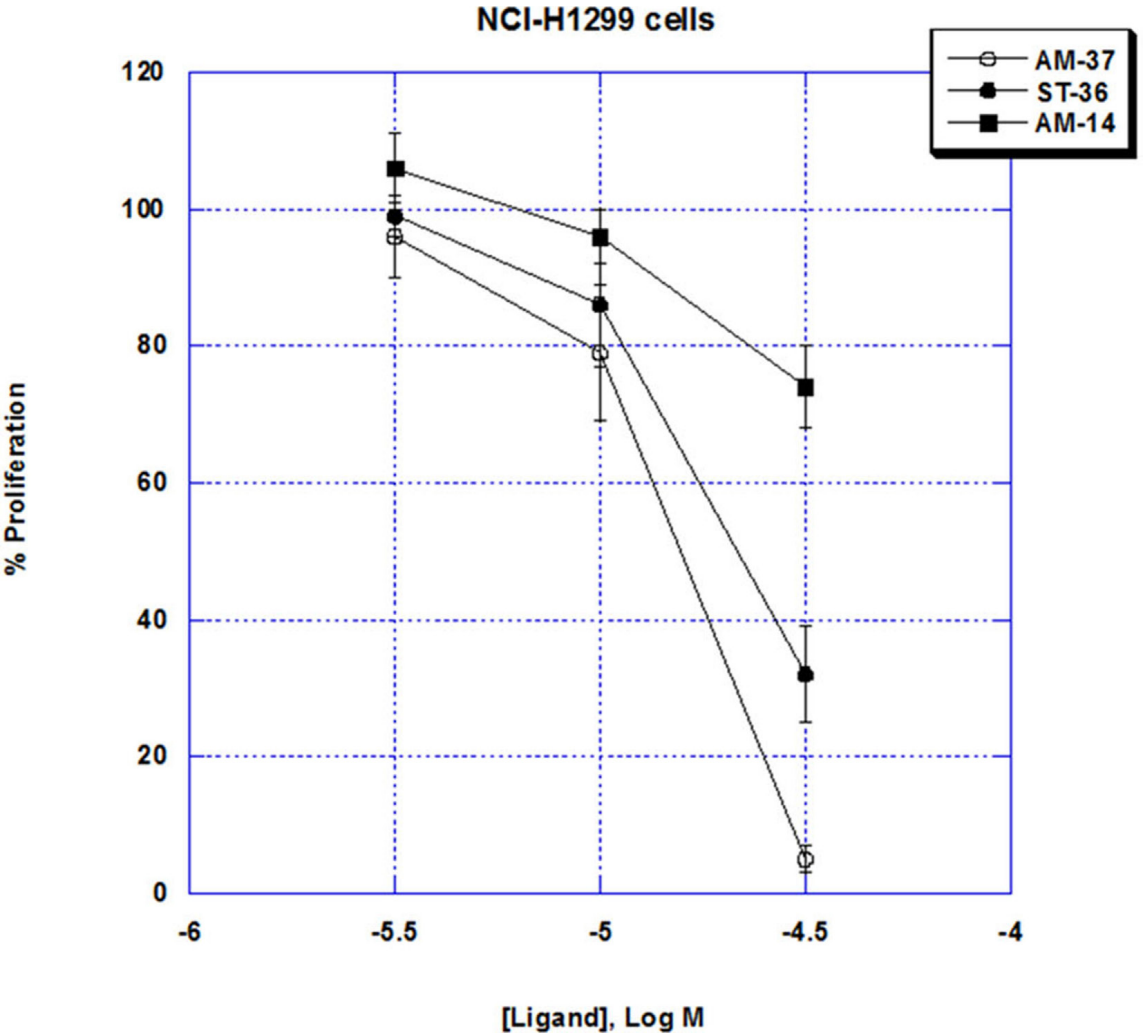
MTT assay. The ability of varying doses of AM-37 (○), ST-36 (●), and AM-14 (■), to inhibit the proliferation of NCI-H1299 cells is shown. The mean value ± SD of eight determinations is indicated. This experiment is representative of two others.

The ability of the small molecules to inhibit lung cancer proliferation was investigated. AM-37 inhibited NCI-H1299 proliferation in a dose-dependent manner. Figure 4 shows that AM-37 had little effect at 3 µM but strongly inhibited proliferation at 30 µM. The IC_50_ for AM-37 was 16 µM. Similarly, ST-36 had an IC_50_ of 22 µM, whereas AM-14 was less potent (IC_50_ > 50 µM).

The specificity of the small molecules was investigated. Table 3 shows that AM-37, ST-36, AM-13, and AM-14 (50 µM) inhibited significantly the proliferation of NCI-H727 cells, which have mRNA for BB_1_R, BB_2_R, and BRS-3. In contrast, AM-37, ST-36, AM-13, and AM-14 had little effect on NCI-H1975 cells, which lack BB_1_R, BB_2_R, and BRS-3. These results indicate that the BBR is essential for AM-37, ST-36, AM-13, or AM-14 to inhibit cancer cellular proliferation.

**Table 3:** MTT proliferation assay using human lung cancer cell lines.

Addition	% proliferation	
	NCI-H727	NCI-H1975
None	100 ± 5	100 ± 6
AM-37, 50 µM	13 ± 1^aa^	103 ± 5
ST-36, 50 µM	30 ± 3^aa^	95 ± 5
AM-13, 50 µM	67 ± 4^a^	89 ± 5
AM-14, 50 µM	64 ± 3^a^	88 ± 6

*The ability of AM-37, ST-36, AM-13, and AM-14 to inhibit non-small cell lung cancer growth was determined using NCI-H727, which express BB_1_R, BB_2_R, and bombesin receptor subtype-3 and H1975 cells, which do not express BBR. The mean value ± SD of eight determinations is indicated. This experiment is representative of two others; ^a^p < 0.05, ^aa^p < 0.01, relative to no additions by ANOVA. Using a test for normality, the data points had a Gaussian distribution*.

## Discussion

While NSCLC patients are traditionally treated with combination chemotherapy, the 5-year survival rate is only 16% (17). Some NSCLC patients (13%) have L858R EGFR mutations, and these patients respond to TKI such as gefitinib or erlotinib; however, secondary EGFR mutations can occur such as T790M resulting in TKI resistance (18). Numerous GPCR are expressed in lung cancer cell lines and biopsy specimens. BB_2_R mRNA is expressed in 46–67% of the lung cancer cell lines examined (19). BB_1_R mRNA is present in 81% of the NSCLC cell lines examined (20). Using autoradiographic techniques, BRS-3 binding sites were detected in 40% of the lung cancer biopsy specimens examined (21). The EGFR is abundant on NSCLC (approximately 100,000 EGFR/cell), whereas BBR are present on most native NSCLC cells (approximately 2,000 BBR/cell) (22).

Addition of GRP to NSCLC cells causes transactivation of the EGFR (23). The effects of GRP on NSCLC tyrosine phosphorylation of the EGFR are impaired by gefitinib, a TKI, and PD176252, a peptoid BB_2_R antagonist. Because the ERK and EGFR tyrosine phosphorylation caused by GRP was impaired by marimastat, GM6001 and antibodies to TGFα, matrix metalloproteases may regulate the cellular shedding of TGFα from NSCLC cells. The TGFα may then bind to the EGFR causing its tyrosine phosphorylation. The results indicate that the BB_2_R regulates EGFR transactivation in NSCLC cells.

The BB_1_R regulates EGFR transactivation (16). The increase in EGFR and ERK tyrosine phosphorylation caused by NMB addition to NSCLC cells was impaired by PD168368, a BB_1_R peptoid antagonist, as well as gefitinib. The increase in EGFR tyrosine phosphorylation caused by NMB was impaired by *N*-acetyl cysteine (NAC), an antioxidant, or tiron, a superoxide scavenger. NMB increased reactive oxygen species (ROS) in NSCLC cells, and the increase was inhibited by Tiron. It remains to be determined if the ROS impair protein tyrosine phosphatases in NSCLC cells, which remove phosphate from the P-EGFR. Activation of BRS-3 with BA1 increased EGFR and ERK tyrosine phosphorylation (13). The increase in EGFR tyrosine phosphorylation caused by BA1 is impaired by NAC, tiron, and diphenyleneiodonium, an inhibitor of NADPH oxidase enzymes.

ML-18 is a small molecule that prefers BRS-3 relative to BB_1_R or BB_2_R (24). ML-18, an S-enantiomer, inhibits ^125^I-BA1 binding to BRS-3, BB_2_R, and BB_1_R with IC_50_ values of 4.8, 16, and >100 μM, respectively, whereas the R-enantiomer EMY-98 is inactive. ML-18 is a BRS-3 antagonist, which inhibits the ability of BA1 to increase cytosolic Ca^2+^, increase ERK and EGFR tyrosine phosphorylation (24). Also, ML-18 inhibited NSCLC growth and increased the cytotoxicity of gefitinib. His^107^ is important for BRS-3 to bind antagonists with high affinity (25). Tyr^101^ of the BB_2_R is important for binding of non-peptide antagonists (26). Similarly, this Tyr is conserved in the BB_1_R and BRS-3. It remains to be determined if this Tyr is essential for binding of AM-37 to the BB_1_R, BB_2_R, or BRS-3. ST-36, which is an S-enantiomer, inhibited specific ^125^I-BA1 binding to BB_1_R, BB_2_R, and BRS-3 with IC_50_ values of 7.9, 6.9, and 10.8 µM, respectively. It is surprising that AM-37, which is the R-enantiomer, binds with slightly higher affinity to BBR than does ST-36. Previously, the BB_1_R was found to prefer PD168,368, which is an S-isomer, relative to the R-isomer (27).

(D-Arg^1^, D-Trp^5,7,9^, Leu^11^)substance P (SP) is an inhibitor of signal transduction and growth of SCLC cells (28). (D-Arg^1^, D-Trp^5,7,9^, Leu^11^)SP impaired the ability of BB, vasopressin, or bradykinin to increase cytosolic Ca^2+^ and ERK activity. (D-Arg^1^, D-Trp^5,7,9^, Leu^11^)SP decreased SCLC growth *in vitro*, and (D-Arg^1^, D-Trp^5,7,9^, Leu^11^)SP has a unique tertiary structure in with two type IV non-standard turns, which juxtapose the N- and C-terminal adjacent to one another (29). Due to this unique structure (D-Arg^1^, D-Trp^5,7,9^, Leu^11^)SP may be able to interact with multiple GPCR. In contrast, AM-37 and ST-36 are small molecules that have a different structure from that of (D-Arg^1^, D-Trp^5,7,9^, Leu^11^)SP.

AM-37 and ST-36 inhibited the proliferation of NSCLC cells such as NCI-H1299 and H727, which have BB_1_R, BB_2_R, or BRS-3. In contrast, AM-37 and ST-36 have little effect on NSCLC cell line NCI-H1975, which lacks BB_1_R, BB_2_R, and BRS-3. It remains to be determined if AM-37 or ST-36 are synergistic with gefitinib at inhibiting the growth of NSCLC. A goal is to identify GPCR antagonists, which potentiate the action of TKI in NSCLC patients.

## Conclusion

AM-37 and ST-36 are small molecules, which bind to the BB_1_R, BB_2_R, and BRS-3. Because AM-37 and ST-36 inhibit the ability of BA1 to increase cytosolic Ca^2+^ as well as increase EGFR and ERK tyrosine phosphorylation, they function as BB_1_R, BB_2_R, and BRS-3 antagonists. A particular advantage of AM-37 and ST-36 is that they will inhibit the growth of NSCLC cells if they have BB_1_R, BB_2_R, or BRS-3.

## Author Contributions

TM and SM were responsible for the receptor binding studies. TM and NT were responsible for the cell culture and calcium experiments. TM, PM, and IR-A were responsible for the transactivation and growth experiments. ML was responsible for the synthesis of the small molecules. TM and RJ were responsible for the writing of the manuscript.

## Conflict of Interest Statement

The authors declare that the research was conducted in the absence of any commercial or financial relationships that could be construed as potential conflicts of interest.
